# Tantalum oxide nanoparticles as versatile and high-resolution X-ray contrast agent for intraductal image-guided ablative procedure in rodent models of breast cancer

**DOI:** 10.1038/s44303-024-00007-5

**Published:** 2024-02-19

**Authors:** Erin K. Zaluzec, Elizabeth Kenyon, Maximilian Volk, Hasaan Hayat, Katherine Powell, Alexander Loomis, Shatadru Chakravarty, Jeremy M. L. Hix, Josh Schipper, Chi Chang, Matti Kiupel, Ping Wang, Erik M. Shapiro, Lorenzo F. Sempere

**Affiliations:** 1 Precision Health Program, Michigan State University, East Lansing, MI 48824 USA; 2 Department of Pharmacology & Toxicology, College of Veterinary Medicine, Michigan State University, East Lansing, MI 48824 USA; 3 Department of Radiology, College of Human Medicine, Michigan State University, East Lansing, MI 48824 USA; 4 Institute for Quantitative Health Science and Engineering, Michigan State University, East Lansing, MI 48824 USA; 5 Van Andel Research Institute, Grand Rapids, MI 49503 USA; 6 Department of Epidemiology and Biostatistics, College of Human Medicine, Michigan State University, East Lansing, MI 48824 USA; 7 Veterinary Diagnostic Laboratory, College of Veterinary Medicine, Lansing, MI 48910 USA; 8 Departments of Biomedical Engineering, Physiology, and Chemical Engineering and Materials Science, Michigan State University, East Lansing, MI 48824 USA

**Keywords:** Cancer, Cancer, Nanoscience and technology, Imaging techniques

## Abstract

There are limited options for primary prevention of breast cancer (BC). Experimental procedures to locally prevent BC have shown therapeutic efficacy in animal models. To determine the suitability of FDA-approved iodine-containing and various metal-containing (bismuth, gold, iodine, or tantalum) preclinical nanoparticle-based contrast agents for image-guided intraductal (ID) ablative treatment of BC in rodent models, we performed a prospective longitudinal study to determine the imaging performance, local retention and systemic clearance, safety profile, and compatibility with ablative solution of each contrast agent. At least six abdominal mammary glands (>3 female FVB/JN mice and/or Sprague-Dawley rats, 10–11 weeks of age) were intraductally injected with commercially available contrast agents (Omnipaque® 300, Fenestra® VC, MVivoTM Au, MVivoTM BIS) or in-house synthesized tantalum oxide (TaOx) nanoparticles. Contrast agents were administered at stock concentration or diluted in 70% ethanol (EtOH) and up to 1% ethyl cellulose (EC) as gelling agent to assess their compatibility with our image-guided ablative procedure. Mammary glands were serially imaged by microCT for up to 60 days after ID delivery. Imaging data were analyzed by radiologists and deep learning to measure in vivo signal disappearance of contrast agents. Mammary glands and major organs were ultimately collected for histopathological examination. TaOx-containing solutions provided best imaging performance for nitid visualization of ductal tree immediately after infusion, low outward diffusion (<1 day) and high homogeneity. Of all nanoparticles, TaOx had the highest local clearance rate (46% signal decay as stock and 36% as ablative solution 3 days after ID injection) and exhibited low toxicity. TaOx-containing ablative solution with 1% EC caused same percentage of epithelial cell death (88.62% ± 7.70% vs. 76.38% ± 9.99%, *p* value = 0.089) with similar minimal collateral damage (21.56 ± 5.28% vs. 21.50% ± 7.14%, *p* value = 0.98) in mouse and rat mammary glands, respectively. In conclusion, TaOx-nanoparticles are a suitable and versatile contrast agent for intraductal imaging and image-guided ablative procedures in rodent models of BC with translational potential to humans.

## Introduction

Breast cancer (BC) has the highest cancer diagnosis rate among women within the United States and is the second leading cause of cancer-related deaths^1^. Although there are many FDA approved treatments for BC patients, the estimated death rate among women has remained stagnant for the past 22 years^1^. Currently, only two FDA-approved options aid in BC prevention: prophylactic mastectomy and hormone therapy^2^. The severe side effects deter many of the eligible women from choosing these interventions^3^. Therefore, there is a need to develop new strategies of prevention for high-risk individuals that will also benefit women at moderate or low risk for BC.

Intraductal injection (ID) can be used clinically for ductography imaging to establish differential diagnosis of nipple discharge with minimal discomfort^4,5^. ID injection is a novel strategy for BC prevention and local treatment^6,7^ that has shown promise in preclinical animal models^8–22^. We have repurposed the procedure of ID injections for BC prevention by infusing a cell-killing solution in rodent models^8–12^, thereby locally targeting epithelial cells from which BC (carcinoma) arises. We previously demonstrated the feasibility of ID delivery with 70% ethanol (EtOH) as an inexpensive, readily available, cell-killing chemical solution in rodent models^8–12^. Our previous study showed therapeutic efficacy of single ID injection of 70% EtOH for preventing BC formation in the aggressive C3(1)-TAg mouse model^8^. As EtOH is already used clinically as an ablative agent for local cancer treatments, these findings position ID injections of 70% EtOH as a promising procedure^10,11^ to investigate in future first-in-human trials for BC prevention of at-risk individuals.

Fluoroscopy and computerized tomography (CT) are clinical imaging modalities that could be used to monitor this ablative procedure for intended application of BC risk reduction in future clinical trials. These techniques are enhanced by contrast agents that attenuate X-rays giving rise to an imaging signal^23^. We seek to introduce a radiopaque contrast agent into our ablative solution for real-time visualization (e.g., fluoroscopy) and image guidance of complete filling of the ductal tree in animal models. MicroCT is a rapid, high-resolution imaging modality that can visualize full anatomy of the breast in animal models in 3D^24^. Several metallic nanoparticle based X-ray contrast agents have been formulated with small nanoparticle size and high radiopacity (reviewed in ref. ^25^). We recently developed tantalum oxide (TaO_x_) nanoparticles as an novel X-ray contrast agent for CT^9^. TaO_x_ nanoparticles have higher radiopacity than iodine at clinical X-ray energies and low toxicity with slower outward diffusion from the ductal tree of rodent models^8–11^ than FDA-approved iodine-based contrast agents (e.g., Isovue-300) used in clinical ductography. Here, we systematically and comprehensively compared the diagnostic and therapeutic potential of several commercially available (iodine-, gold-, and bismuth-containing) X-ray contrast agents against TaO_x_ nanoparticles when infused intraductally with the 70% EtOH ablative solution. We set the following criteria to objectively identify suitable contrast agent for image guidance of this investigational ablative procedure: (1) minimal diffusion within 8 h of infusion to have a nitid visualization and assess complete filling of the ductal tree immediately after injection; (2) high local clearance by 3 days, so that contrast agent does not interfere with future imaging sessions (CT and/or MRI) assessing anatomical changes of treated mammary gland; (3) low local and systemic toxicity; and (4) compatibility with ablative agent (70% EtOH) to maximize epithelial cell killing.

## Materials and methods

### Contrast agent preparation

All X-ray contrast agents were used as commercially supplied. Hydrophilic TaOx nanoparticles were synthesized as described^9^. For all contrast reagents except TaO_x_ nanoparticles (NPs), injection solution was either “stock” as supplied by the manufacturer with no dilution, or three parts stock solution with 7 parts 200 proof EtOH to yield 70% EtOH in the solution: Omnipaque® 300 (GE Healthcare, #00407141363, 300 mg I/ml), Fenestra® VC (MedLumine, VC-131, <200 nm particles pegylated emulsion, 50 mg I/ml), MVivo^TM^ Au (MedLumine, Au-315, 15 ± 2 nm particles, 200 mg Au/ml), MVivo^TM^ BIS (MedLumine, BIS-11, 250 nm nanoparticles, 150 Bi mg/ml). TaO_x_ (11.1 ± 1 nm particles) was supplied in lyophilized form as described^9^, which allowed for the 70% EtOH solution to contain half the concentration of the “stock” solution rather than 30% as with manufactured contrast agents (specifically 36 mg Ta/ml in “stock” versus 18 mg Ta/ml in 70% EtOH). Ethyl cellulose (Acros Organics, 9004-57-3) was added up to 1% (w/v) to 70% EtOH solution containing 18 mg Ta/ml of TaO_x_.

### microCT image acquisition and analysis

**Fig. 1:**
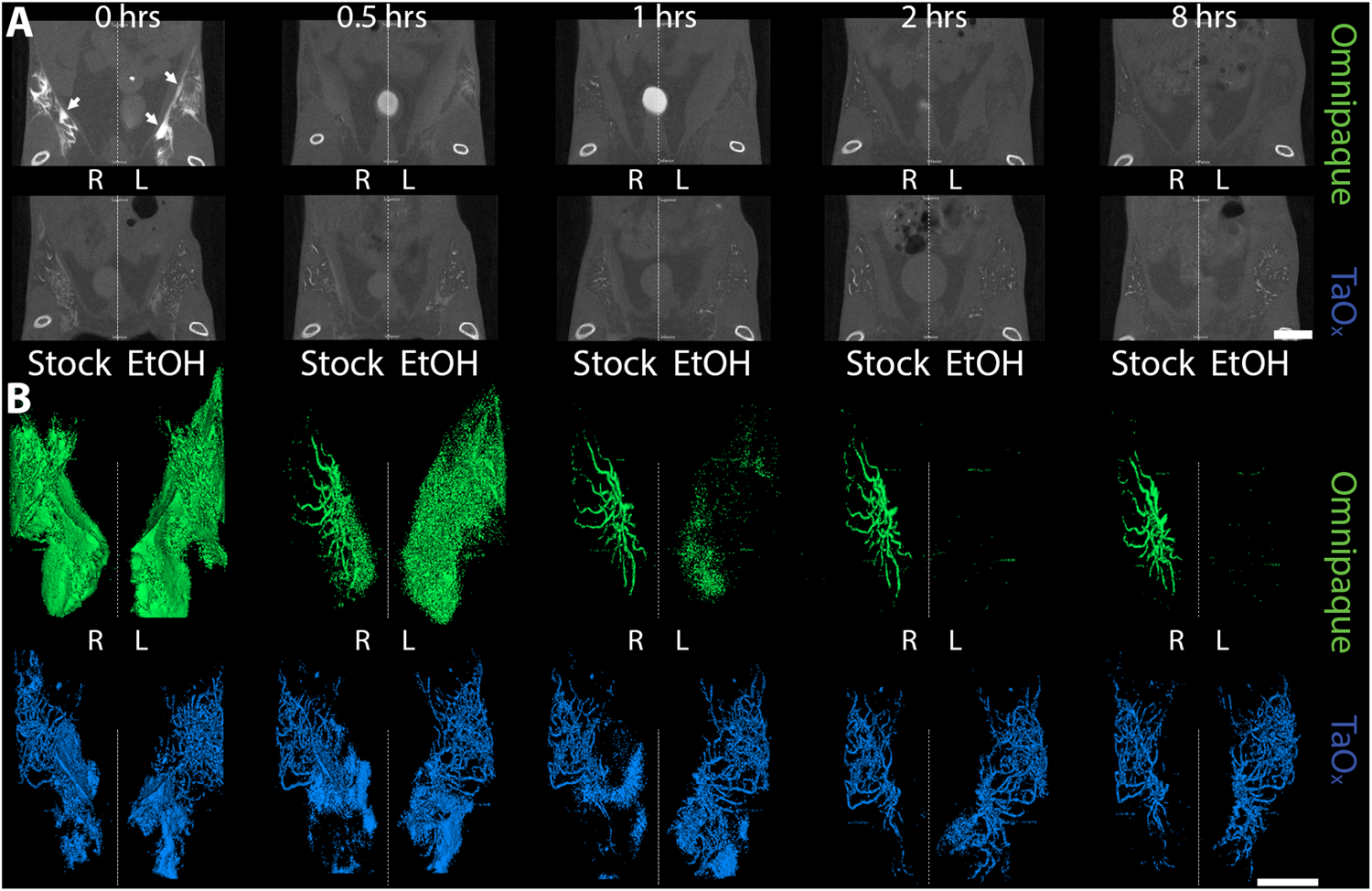
Short-term serial microCT imaging of the murine ductal tree with different contrast agents. Abdominal mammary glands were injected with 40 μl of indicated contrast agent as stock solution (300 mg I/ml Omnipaque or 18 mg Ta/ml in PBS) or in 70% ethanol (EtOH, 90 mg I/ml Omnipaque or 18 mg Ta/ml). **A** Representative microCT slice of the lower body of the same animals is shown at different imaging time points from immediately after last ID injection (0 h) to 8 h. Scale bar is 10 mm. **B** 3D reconstruction of manually segmented region of interest (i.e., ipsi- and contralateral abdominal mammary glands). 3D reconstruction was thresholded to include only voxels with a HU value of >300. Arrows indicate areas in which leaked contrast agent accumulates on the fascia boundary. Scale bar is 1 mm.

All experiments were conducted under protocols approved by Institutional Animal Care and Use Committee at Michigan State University. Ten-week-old female FVB/JN mice (*n* = 3–5/solution; jax.org stock 001800) and 11-week-old female Sprague Dawley rats (*n* = 3–4/solution; envigo.com order code 002) were prepared and ID injected as described^10,11^. Serial images of infused ductal trees were acquired at different time points post-injection using a PerkinElmer Quantum GX microCT scanner; for short-term study: 0, 30 min, 1, 2, 4 and 8 h (Figs. 1 and S1) and for long-term study: 0, 1, 3, 7, 14, 30 and 60 days (Figs. 3, 4 and S2). The following image acquisition scan parameters were standardized and used at each scan interval time point in mice: 90 kVp/88 μA; field of view (FOV), 36 mm; number of slices, 512; slice thickness, 72 μm; voxel resolution, 72 μm^3^; and in rats: 90 kVp/88 μA; FOV, 72 mm; number of slices, 512; slice thickness, 72 μm; voxel resolution, 144 μm^3^. Radiation exposure was minimized in these serial imaging studies by acquisition of standard (2 min) scans. Caliper AnalyzeDirect©, v12.0 (Biomedical Imaging Resource, Mayo Clinic, Rochester, MN) was used for microCT image rendering, segmentation, and analysis of individual glands or tissue phantoms (0.2 ml PCR tubes with contrast solutions) as described^10,11^.

### microCT deep learning analysis

A deep learning algorithm consisting of a convolutional neural network (CNN) with UNET architecture was used for automated segmentation and analysis of the microCT images. Two clinical radiologists (A.L, P.W.) and an imaging specialist (H.H.) generated full volume segmentation masks for 12 image volumes consisting of 256 slices each via the ITK-snap software. The model consisted of feature extraction, flattening and regression layers, taking the preprocessed image as input. The model was trained using individual 2D slices and corresponding radiologist generated masks from each image volume, which rendered a total dataset size of 3072 slices used for initial training of the algorithm. Preprocessed image slices of dimensions [x, y] = [256, 512] were input into the algorithm for training with a batch size = 32, epoch = 100 iterations and a learning rate *α* = 10^-4^ using the Adam optimizer. Loss per iteration was calculated using standard gradient descent loss algorithm^26^. A 5-fold cross-validation method was used for training such that 80% of the data was used as a training set and 20% of the data was used for internal validation of algorithm performance. The resulting image segmentations were then used for ROI analysis of mammary gland contrast content using average HU values rendered from each gland bilaterally. For adequate transformation of pixel values to standard Hounsfield units (HU), the inference script used SimpleITK for rendering of the original image and resulting segmentation output from the algorithm after thresholding the original image using the ROI prediction mask. The inference script did not threshold images at a specified HU value.

### Histological analysis

Animals were euthanized immediately after last scan in serial microCT imaging (for short term study 8 h after and for long term study 60 days after injections). Dissected mammary glands were processed and embedded in paraffin after 24 h fixation in formalin as described^27^. Formalin-fixed paraffin-embedded (FFPE) tissue samples (4 µm) were scanned on an Aperio Versa 8 Brightfield&Fluorescence imaging system (Leica Biosystems, Buffalo Grove, IL) following H&E staining. Annotation and quantitative analysis were performed using ImageScope tools as described^8^.

### Statistical analysis

Unpaired Welch’s *t*-tests were used to assess statistical significance of difference of continuous values obtained from imaging and tissue analyses between experimental groups and a reference control group. GraphPad Prism 9 was used to perform these statistical analyses. We set a *p* value of 0.01 as the threshold to report statistical significance.

## Results

### TaO_x_ enables better imaging of rodent ductal tree architecture as compared to FDA approved Omnipaque

We conducted a short-term serial imaging study to characterize retention of Omnipaque and TaO_x_ within the ductal tree. We injected 40 µl of Omnipaque or TaO_x_ in PBS (300 mg I/ml and 18 mg Ta/ml, respectively) or 70% EtOH (90 mg I/ml and 18 mg Ta/ml respectively) in the abdominal mammary glands of FVB mice. Animals were imaged by microCT immediately and at 0.5, 1, 2, 4 and 8 h post injections (Fig. 1A). Stock Omnipaque was detected at all time points, but rapidly diffused outside the ductal tree, flooding the mammary fat pad and reaching the fascia (Figs. 1A, B and S1). After local clearance, residual Omnipaque was retained within the ductal tree enabling nitid visualization of its overall structure from 1 to 8 h after injections. Omnipaque diluted in 70% EtOH had minimal retention in the ductal tree and was undetectable 1 h after injections (Figs. 1B and S1). However, TaO_x_ remained within the ductal tree with little clearance and had extended ductal tree branching within the 8-h time frame in both stock PBS solution and 70% EtOH (Figs. 1A, B and S1). Most importantly, TaO_x_ enables nitid visualization of the ductal tree immediately after infusion with 70% EtOH ablative solution, which is a required feature for intended image guidance application to assess complete filling of the ductal tree. Together, these demonstrate TaO_x_ has superior local retention and imaging capabilities as compared to Omnipaque.

### In vitro and in vivo comparison of TaO_x_ with commercially available CT contrast agents

**Fig. 2:**
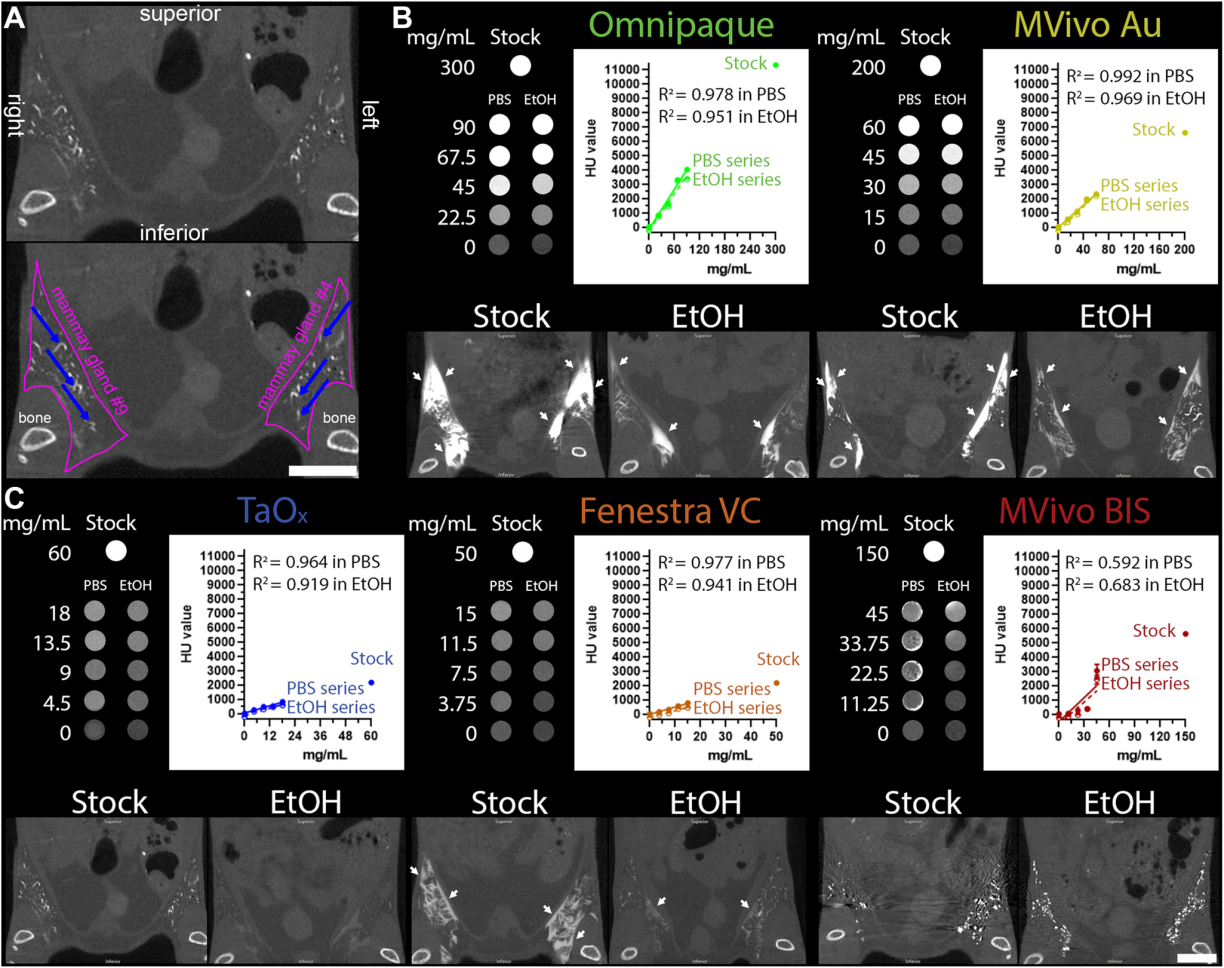
Contrast agent characteristics and signal attenuation profile in different solutions. **A** Annotated views of microCT image of TaO_x_-infused mammary glands (36 mg Ta/ml stock, also shown at lower magnification in **C**); pink line outlines abdominal mammary glands and blue arrows pinpoint filled branches of the ductal tree. **B**, **C** Tissue phantoms and mice were scanned with the same microCT imaging parameters. Top panels (tissue phantoms), each contrast agent was diluted from stock reagent (maximal concentration) in PBS or 70% ethanol (EtOH) at indicated concentrations (mg of metal/ml). Linear fitting of signal attenuation as function of the concentration of the metal in each solution. Bottom panels, representative single-slice microCT images of the lower body of animals captured immediately after last ID injection of each indicated solution: Omnipaque (300 mg I/ml stock, 90 mg I/ml in EtOH), MVivo Au (200 mg Au/ml stock, 60 mg Au/ml in EtOH), TaO_x_ (36 mg Ta/ml stock, 10.8 mg Ta/ml in EtOH), Fenestra VC (50 mg I/ml stock, 15 mg I/ml in EtOH), MVivo BIS (150 mg Bis/ml stock, 45 mg Bis/ml in EtOH). Arrows indicate areas in which leaked contrast agent accumulates on the fascia boundary. Scale bar is 10 mm in images at different magnification.

To ensure that all contrast agents could be visualized by microCT imaging at the injectable range, stock solutions of each agent were serially diluted in PBS or 70% EtOH (Fig. 2). Omnipaque and Mvivo Au dilutions had the highest signal intensity, though all contrast agents produce adequate signal. EtOH did not interfere with signal detection or homogeneity of any of the contrast agent, except for MVivo BIS (Fig. 2B, C). Qualitatively, MVivo BIS signal was heterogenous in tissue phantom. Quantitatively, linear fitting of MVivo signal was poor in PBS (*R*^2^ = 0.59 compared to other contrast agents *R*^2^ > 0.96) and in 70% EtOH (*R*^2^ = 0.68 compared to other contrast agents *R*^2^ > 0.91). Commercial contrast agents were provided at stock concentration ready to inject intravenously in animals. Therefore, the addition of EtOH resulted in lowered concentration of each contrast agents to 30% of maximal concentration, except for TaO_x_ nanoparticles that can be reconstituted to up to 60 mg Ta/ml in either PBS or 70% EtOH (Fig. S2). As expected, stronger signal can be observed in infused ductal tree with stock solution rather than EtOH. The decrease of signal intensity of all contrast agents is proportional and consistent with observations in tissue phantoms, except for MVivo BIS (Fig. 2B, C). As observed in tissue phantoms, MVivo BIS signal was inconsistent and heterogeneous within the ductal tree. Moreover, viscous, aggregation-prone MVivo BIS solutions difficulted a steady continuous infusion of cannulated nipples.

**Fig. 3:**
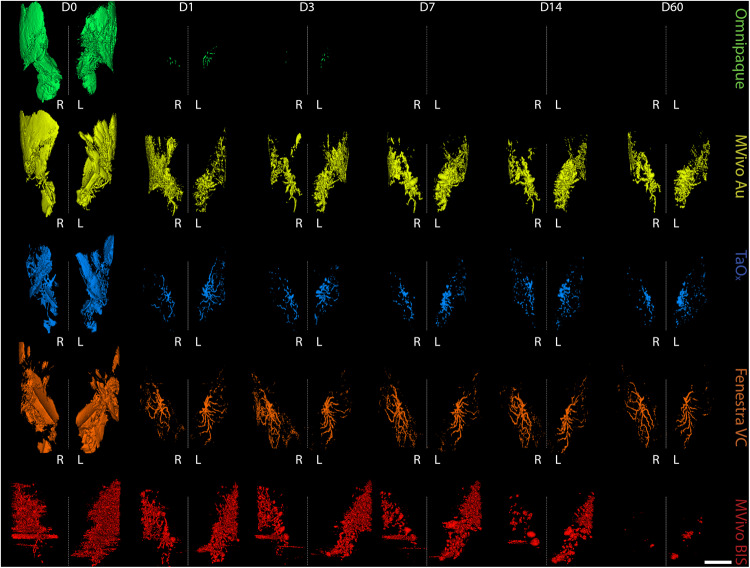
Long-term serial microCT imaging of the murine ductal tree with different contrast agents in stock solution. Abdominal mammary glands were injected with 40 μl contrast agent as stock solution as indicated: Omnipaque (300 mg I/ml), MVivo Au (200 mg Au/ml), TaOx (36 mg Ta/ml), Fenestra VC (50 mg I/ml), MVivo BIS (150 mg Bis/ml). 3D reconstruction of manually segmented regions of interest (i.e., ipsi- and contralateral abdominal mammary glands) of the same animals is shown at different imaging time points from immediately after (D0) last ID injection to 60 days (D60). 3D reconstruction was thresholded to include only voxels with a HU value of >300. Scale bar is 1 mm.

**Fig. 4:**
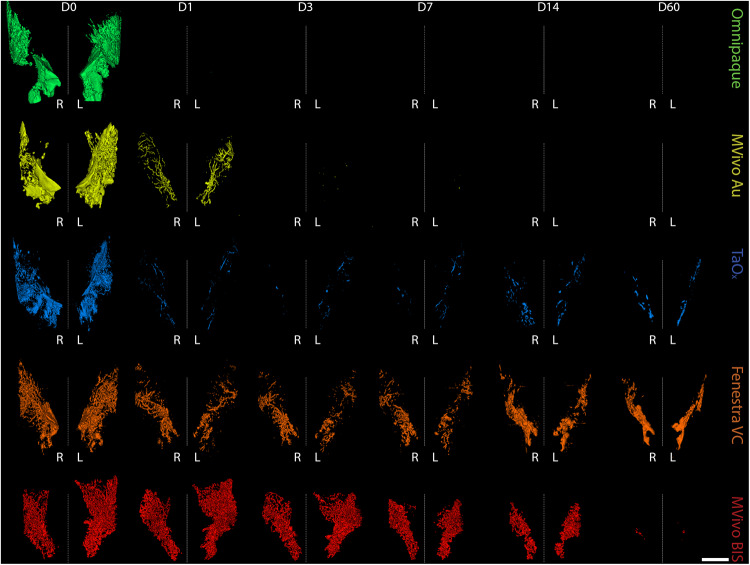
Long-term serial microCT imaging of the murine ductal tree with different contrast agents in 70% ethanol. Abdominal mammary glands were injected with 40 μl of indicated contrast agent in 70% ethanol: Omnipaque (90 mg I/ml), MVivo Au (60 mg Au/ml), TaO_x_ (10.8 mg Ta/ml), Fenestra VC (15 mg I/ml), MVivo BIS (45 mg Bis/ml). 3D reconstruction of manually segmented regions of interest (i.e., ipsi- and contralateral abdominal mammary glands) of the same animals is shown at different imaging time points from immediately after (D0) last ID injection to 60 days (D60). 3D reconstruction was thresholded to include only voxels with a HU value of >300. Scale bar is 1 mm.

From the short-term serial imaging study (Fig. 1), we determined that the time point immediately after injection was crucial for the ability of a contrast agent to serve in image guidance application of this ablative procedure. To assess imaging performance of all contrast agents for initial visualization of the ductal tree, we generated 3D reconstructions of the infused ductal trees from segmented images of the mammary gland structures (fat pad/fascia boundary) (Fig. 2A). Omnipaque and Mvivo Au solutions rapidly diffused outside the ductal tree and flooded mammary gland stroma as inferred by the oversaturation and lack of defined ductal tree structure (Figs. 3, 4 and S2). Leaked contrast agent can be easily appreciated as it markedly outlines the mammary gland fascia boundary on single-slice microCT images (Fig. 2B) and a solid wall on 3D reconstructions (Figs. 3 and 4). Fenestra VC and TaO_x_ solutions were predominantly retained within the filled ductal tree enabling informative visualization of the overall ductal tree structure (Figs. 2C, 3, 4 and S2). Compared to TaO_x_, the ductal tree in Fenestra VC-infused animals was equivocal and not as defined, especially in stock solution, due to local leakage outside the tree and heterogenous distribution of the solution (e.g., air bubbles) (Figs. 2C, 3, 4 and S2).

### Local retention and long-term imaging of residual contrast agents

To study the long-term effects of local retention and systemic clearance of each contrast agent, we conducted a 60-day serial imaging study. Mice were ID infused with 40 µl of stock solutions or contrast agent in 70% EtOH into the abdominal mammary glands. Animals were imaged by microCT at days 1, 3, 7, 14, 30 and 60 after injections (Figs. 3, 4 and S2). We generated 3D reconstructions of the lower body to determine how contrast agents distributed systemically. We did not detect signal for any contrast agents in major organs (kidney, lung, liver, spleen) (Fig. S2). Mvivo Au solutions accumulated subcutaneously (Fig. S2). Subcutaneous accumulation was occasionally observed in animals infused with TaO_x_ (1 out of 15 animals) or Fenestra VC (1 out of 7) stock solutions (Fig. S2). We generated 3D reconstructions of the infused ductal trees from segmented images of the mammary gland structure (fat pad/fascia boundary). As expected after day 1, Omnipaque-infused animals had little signal retention in the mammary gland compared to the other contrast agents (Figs. 3 and 4). Mvivo Au, TaO_x_, and Fenestra VC remained within the ductal tree for 60 days to varying degrees (Figs. 3 and 4), except for Mvivo Au in 70% EtOH which was undetectable after 7 days (Fig. 4). Fenestra VC in 70% EtOH appeared to aggregate during the active process of wound healing, hampering local clearance (Fig. 3). Further study will be needed to understand if this aggregation is a macrophage-mediated process or another foreign object clearance mechanism is at work. The faster clearance and low immunogenicity of TaO_x_ is a desirable feature to minimize long-term toxicity and facilitate follow-up procedures.

### AI-assisted quantitative metrics of contrast agent signal decay

**Fig. 5:**
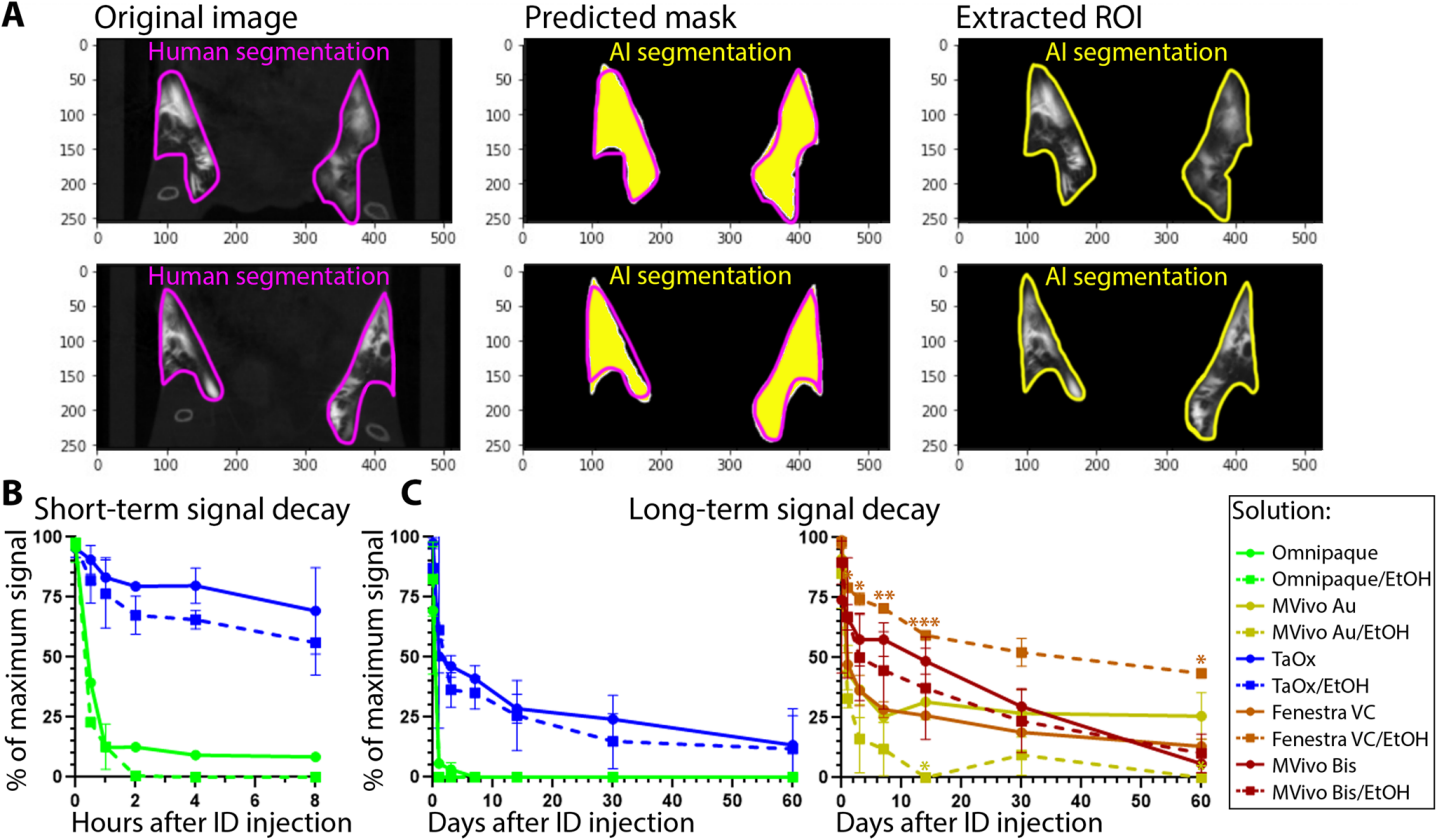
AI assisted quantitation of signal decay and local clearance of each contrast agent. **A** A deep learning algorithm was used to train gland segmentation using mask parameters defined by radiologists; Original, representative microCT image slices of contrast agent-injected mammary glands, radiologist-labeled segmentation masks per slice, AI prediction of segmentation masks per slice, automated AI segmentation result for image slice. **B** AI-assisted quantification of signal decay in short-term serial imaging characterization of indicated solutions (as shown in Fig. 1). **C** AI-assisted quantification of signal decay in long-term serial imaging characterization of indicated solutions (as shown in Figs. 3 and 4). Asterisks indicate *p* value of unpaired Welch’s *t*-test of stock compared to 70% EtOH solution of each contrast agent per time point (* <0.01, ** <0.001, *** <0.0001).

To obtain systematic and quantitative metrics of signal decay over time, we developed a Deep Learning (DL)-based AI algorithm for automated segmentation of mammary gland and extraction of HU values from region of interest (Fig. 5A). We applied this DL algorithm for image analysis of data filles obtained from short-term study (Fig. 1) and long-term study (Figs. 3 and 4). Omnipaque signal was less than 15% of maximum signal in either stock or 70% EtOH after 1 h of injection (Fig. 5B) and signal was less than 5% after 1 day and undetectable after 3 days (Fig. 5C). TaO_x_ signal remained above 60% of maximum both in stock and 70% EtOH solution after 8 h of injections (Fig. 5B). After 1 day of injections, TaO_x_ signal was less than 40% of maximum and exhibited more rapid local clearance than the other nanoparticle-based contrast agents, except for MVivo Au in 70% EtOH (Fig. 5C). TaO_x_ signal was less than 20% of maximum by 60 days after injections. Comparatively, Mvivo Bis signal declined more slowly during the first 30 days after injections, but precipitously declined to less than 10% by 60 days (Fig. 5C). Unlike other contrast agents, Fenestra VC signal in 70% EtOH declined much slower than in stock solution and almost 50% of signal remained 60 days after injections (Fig. 5C).

### Ductal ablation, systemic accumulation and toxicity of TaO_x_ and other contrast agents

**Fig. 6:**
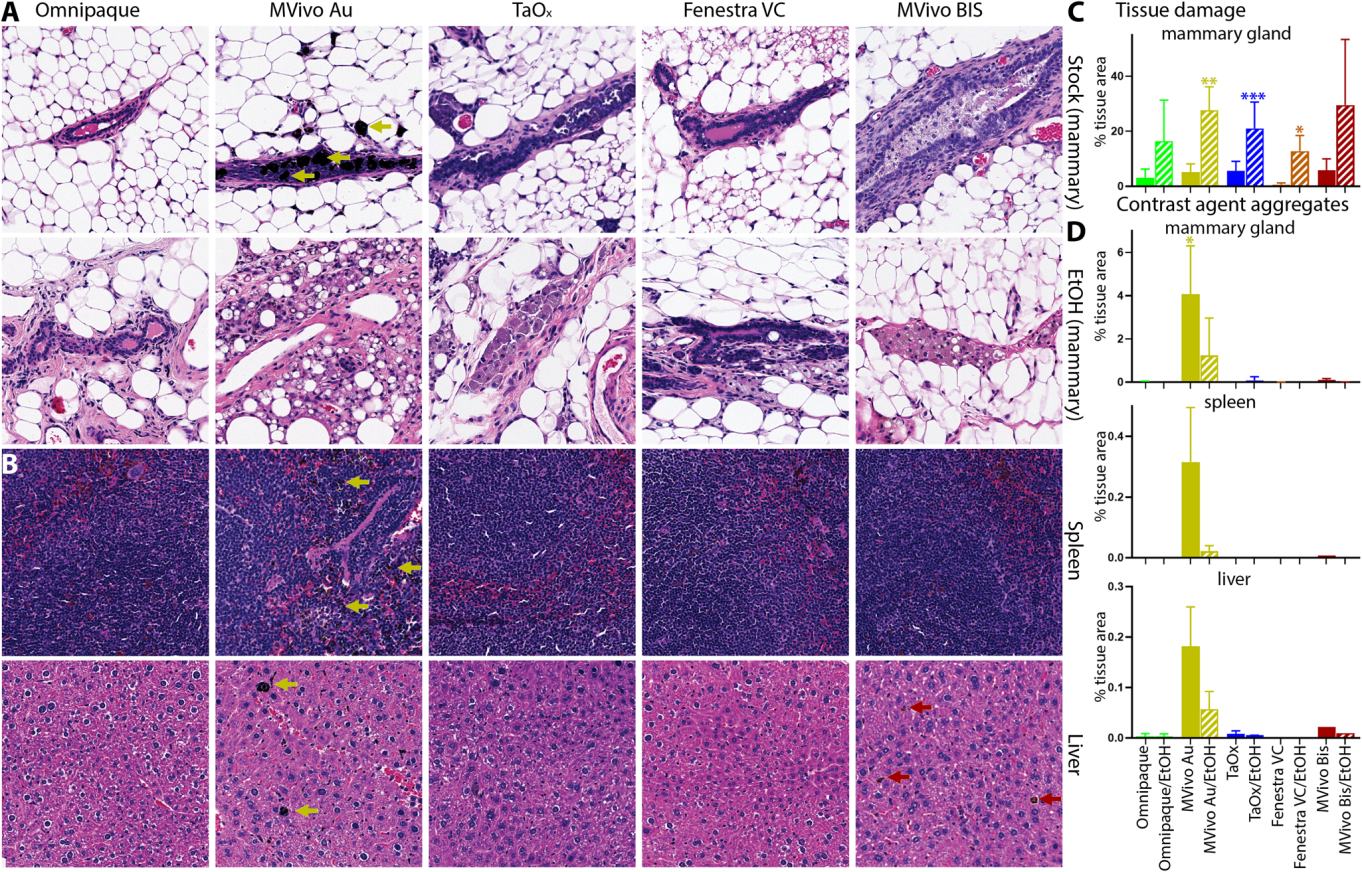
Local and systemic clearance of contrast agents in different solutions. Representative H&E staining of the mammary gland, spleen, and liver 60 days after ID injection of indicated contrast agent as stock solution (**A**, **B**): Omnipaque (300 mg I/ml), MVivo Au (200 mg Au/ml), TaOx (36 mg Ta/ml), Fenestra VC (50 mg I/ml), MVivo BIS (150 mg Bis/ml), or in 70% ethanol (EtOH) (**A**): Omnipaque (90 mg I/ml), MVivo Au (60 mg Au/ml), TaOx (10.8 mg Ta/ml), Fenestra VC (15 mg I/ml), MVivo BIS (45 mg Bis/ml). Arrows point to nanoparticle aggregates. **C** Morphology-driven quantitation of tissue damage, which includes fibrosis, inflammation and scarring resulting from ablative effects of 70% EtOH as well as immune cell-mediated foreign object reaction to clear nanoparticle-based contrast agents. **D** Quantitation of visually apparent aggregates of nanoparticle-based contrast agents in indicated tissues. Asterisks indicate *p* value of unpaired Welch’s *t*-test of each solution compared to Omnipaque stock (* < 0.01, ** <0.001, *** <0.0001).

To ensure the safety of the contrast agents for application in the ablative procedure, we next looked at local and systemic toxicity of each contrast agent alone or in combination with 70% EtOH. Tissues from mammary glands and major organs (heart, lung, spleen, liver, and kidney) were collected immediately after last imaging session 60 days after injections. For all contrast agents, we observed healthy, nucleated epithelial cells with intact surrounding adipose tissue in H&E stained mammary glands (Fig. 6A). A mild foreign body reaction with periductal fibrosis was observed in MVivo Au- and TaOx-infused ductal trees; a stronger foreign body reaction with periductal fibrosis and inflammation as well as intraductal histocyte accumulation was observed in MVivo BIS-infused ones (Fig. 6A). Contrast agents had no or minimal interference with ablative effects of 70% EtOH and wound healing response. A similar amount of tissue damage was observed in all tested contrast agent conditions in 70% EtOH (Fig. 6A, C). Interestingly, pockets of intact epithelial cells were observed in Omnipaque- and Fenestra VC-infused ductal tree, suggesting an incomplete epithelia ablation perhaps due to uneven and heterogenous distribution throughout the lumen of all branches (Fig. 6A). Accumulation of nanoparticle aggregates was only visible in MVivo Au-infused ductal trees (Fig. 6A, D); these aggregates were also observed in distant organs, especially in the spleen and liver (Fig. 6B, D). However, no overt toxicity was observed in major organs whether there were visible nanoparticle aggregates (MVivo Au) or not; there were no atypical tissue presentations in the form of dysplasia, infarction, hemorrhage, fibrotic or immune reaction (Fig. 6B).

### Compatibility with ethyl cellulose and scalability of TaO_x_-based ductal tree visualization in rats

**Fig. 7:**
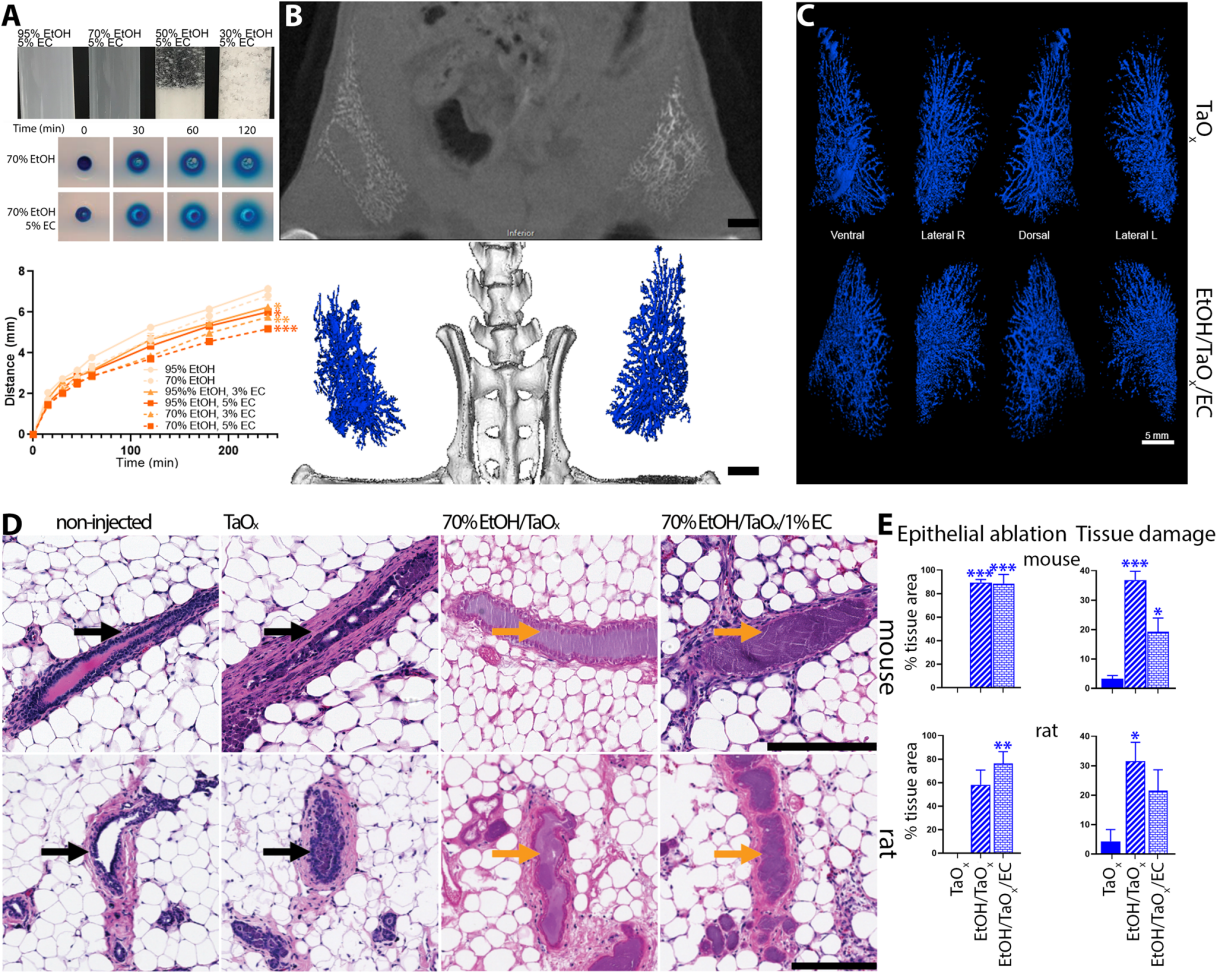
Compatibility and scalability of TaO_x_-containing solutions. **A** Indicated blue dye-containing solutions were dispensed into 1% agarose-casted 5-mm circular cylindrical channels (tissue phantoms). The distance the blue dye front traveled from the edge of each channel (*x*) was plotted over time (*t*). All solutions fit (*R*^2^ > 0.99) Fick’s equation *x* = (4*Dt*)^½^, where *D* is the diffusion constant. Asterisks indicate *p* value of unpaired Welch’s *t*-test of each solution compared to 95% EtOH (* < 0.01, ** <0.001, *** <0.0001). **B**, **C** Ductal trees of abdominal mammary glands were infused with 250 μl of indicated TaO_x_-containing solutions (18 mg Ta/ml). **B** Representative microCT slice of the lower body of an animal is shown immediately after last ID injection. Scale bar is 10 mm. **C** 3D reconstruction of manually segmented mammary gland per condition is shown at different views. 3D reconstruction was thresholded to include only voxels with a HU value of >300. Scale bar is 5 mm. **D** Representative H&E staining of mouse and rat mammary gland 3 days after ID injections of indicated TaO_x_-containing solutions (18 mg Ta/ml). Intact (black arrow) and ablated ducts (orange arrow) are indicated. Scale bar is 200 μm in images at different magnification. **E** Morphology-driven quantitation of epithelial ablation (anucleate cells, cytoplasmic hypochromia) and tissue damage, which includes fibrosis, inflammation and scarring resulting from ablative effects of 70% EtOH as well as immune cell-mediated foreign object reaction to clear nanoparticle-based contrast agents. Asterisks indicate *p* value of unpaired Welch’s *t*-test of each solution compared to TaO_x_ solution.

Together, the above results support the superiority of TaO_x_ as contrast agent for this ablative procedure. TaO_x_ was retained in the ductal tree and did not cause toxic effects locally or systemically. TaO_x_ did not interfere with EtOH ablative effect nor were TaO_x_ imaging properties impacted by EtOH. Therefore, our expansion studies exclusively focused on the ability of TaO_x_ to be formulated in solution with ethyl cellulose (EC) as gelling agent and the ability of this refined solution and ablative procedure to be scaled up to a rat model. EC is used clinically in 95–100% EtOH solution for ablative treatment of tumors and sclerosing treatment of venous malformations^28–35^. We first assessed the ability of EC to slow down EtOH diffusion using tissue phantoms. EC at 5% w/v concentration is not soluble in less than 70% EtOH because of the increased water content (Fig. 7A). Compared to other tested solutions, 70% EtOH with 5% EC had the lowest rate of diffusion (Fig. 7A). To determine the ability of EC to limit EtOH diffusion throughout the mammary gland in vivo, the ductal trees of both mice and rats were infused with a 70% EtOH solution containing TaO_x_ (18 mg Ta/ml) and/or EC (1% w/v). ID injections were successfully translated into the rat model and X-ray imaging capabilities of TaO_x_ were maintained for visualization of infused rat ductal trees (Fig. 7B, C). Macroscopically, EC-containing solutions provide same infusion properties and ductal tree visualization as undoped solutions (Fig. 7C). Microscopically, EC-containing solutions provide same or higher epithelial cell ablation rate and significantly lower collateral tissue damage both in mouse and rat mammary gland tissues examined 3 days after ID injections (Fig. 7D, E). Together, these results indicate that introducing 1% EC to ablative and imaging solution of 70% EtOH and TaO_x_ (18 mg Ta/ml) further improves local targeting epithelial cells with less collateral tissue damage.

## Discussion

We evaluated the short-term and long-term performance of contrast agents in visualizing the infused ductal trees in rodent models and any impact on ablation rate, breast physiology, and scalability to larger animal models and eventually humans. From a clinical standpoint, fluoroscopy or similar real-time imaging modality will be needed to guide the ductal tree infusion in future first-in-human clinical trials to evaluate this ablative procedure. Given the desired properties of an ideal contrast agent (visualization of fully filled ductal tree, high local clearance, low toxicity, and compatibility with 70% EtOH) for this therapeutic purpose, we identified TaO_x_ as the most suitable contrast agent. Rapid outward diffusion, especially in 70% EtOH, of FDA-approved Omnipaque (Figs. 1–4 and S1, S2) and gold nanoparticle-containing MVivo Au (Figs. 2–4 and S2) renders them unsuitable for the intended image guidance application of assessing fully filled ductal tree(s). Omnipaque presumably escapes the ductal tree system after extensive epithelial cell ablation and loss of architectural integrity with hyperintensity in the bladder from 0.5 to 2 h indicating rapid systemic clearance (Fig. S1), but additional experiments are needed to test this directly. An unexpected concern of MVivo Au was the discoloration of mice. Immediately after injections, all mice turned a gray color which did not resolve throughout the study. Although there was no observed discomfort or overt toxicity, the systemic spread to internal organs (Fig. 6) and subcutaneous accumulation (Fig. S2) is a cause for concern for continued use. Other contrast agents (Fenestra VC and MVivo BIS) and TaO_x_ exhibited a much higher local retention (Fig. 5B) and enabled initial visualization of infused ductal trees (Figs. 2–4 and S2). However, MVivo BIS produced imaging artifacts that compromised unequivocal and nitid visualization of the true ductal tree architecture (Figs. 2–4 and S2). Fenestra VC enabled similar short-term visualization of the ductal tree as TaO_x_, with some imaging artifacts due to heterogenous dispersion and diffusion through the lumen and leakage outside the ductal tree (Figs. 2–4 and S2). Higher local retention and limited clearance of Fenestra VC, especially in 70% EtOH, is a problematic feature of this contrast agent (Figs. 4 and S2). In exploratory experiments, animals infused with Fenestra VC or MVivo BIS in more than two mammary glands died shortly after injections. The total amount of contrast injected was above vendor’s recommended bolus dose for intravenous administration. This suggests that the maximal tolerated dose of these contrast agents as formulated would be a limiting factor for intended application in ID imaging procedures in women. In contrast, animals infused with TaO_x_ in six or more mammary glands tolerated this procedure well^10,11^.

A main goal of this study was the serial imaging of different contrast agents. This required several sessions with X-ray radiation. While the cumulative X-ray dose delivered was less than 500 mGy^36^, radiation may have contributed to foreign object recognition and clearance by the host immune system and overall wound healing process. Short-term effects of radiation (<7 days after injections) did not appear to interfere with EtOH-induced epithelial ablation with different contrast agents compared to EtOH treatment alone in our previous study^8^. Long-term effects of radiation and/or contrast agent additions appeared to delay wound healing process compared to EtOH treatment alone^8^. We used commercially available contrast agents at stock concentrations recommended by the vendor for intravenous injections. We acknowledge that refinement of the concentration or formulation of these nanoparticle-based contrast agents may make them more suitable for the intended intraductal applications, especially since many of these agents are for preclinical research purposes only. However, under same conditions, off-the-shelf TaO_x_ nanoparticles outperformed all these contrast agents. This highlights the versatility of our in-housed synthesized TaO_x_ construct that can be reconstituted at a wide range of concentrations in hydrophilic, polar, and hydrophobic solutions (Fig. S2, ref. ^9^).

We also broadened the versatility of TaO_x_ as imaging agent by scaling up ID procedure and in vivo X-ray visualization of the infused ductal tree in rat models (Fig. 7B, C). We introduced ethyl cellulose (EC) as gelling agent to limit collateral tissue damage caused by diffusion of EtOH. EC is safe for human consumption and is clinically used with EtOH for treatment of tumors and venous malformation^28–35^. The addition of 1% EC had no impact on ablative rate and aided in reducing EtOH dispersion outside of the ductal tree (Fig. 7D, E). However, some animals experienced limb stiffness with the addition of EC. Tissue analyses show wound healing process resolves about 1 month after ablative procedure (Fig. 7A). However, further investigation will be needed to determine the specific immunological and fibroblastic responses to tissue damage that may be caused and compounded by the combination of 70% EtOH, TaO_x_ and/or EC, and if higher % of EC may be beneficial to faster resolution of wound healing. Current protocols for BC diagnosis utilize X-ray or MR imaging for confirmation of masses within the breast and lack of clearance of a contrast agent might interfere or create imaging artifacts. While TaO_x_ signal is less than 36% of maximal signal 3 days after injection, there is still about 10% of maximal signal detected after 60 days (Fig. 5B). Therefore, it will be important to refine TaO_x_ formulation to maximize clearance after 3 days and/or determine what amount of residual contrast agent may have a potential impact on follow-up imaging session for anatomical assessment and/or tumor surveillance. While these rodent models are well-established for assessing therapeutic efficacy (tumor latency, tumor incidence, and overall survival), both mice and rats have a single-ductal tree per mammary gland with a relatively simple and linear structure^37,38^. Rabbits are a larger animal model closer to humans evolutionarily, physiologically, and anatomically with multiple ducts per mammary gland^39–46^. Therefore, utilizing rabbit models and fluoroscopy to guide infusion of cannulated nipples in future studies should improve the success rate of the procedure, address challenges of simultaneous infusion of multi-ductal tree system and impact on cosmesis and collateral tissue damage, and further assess the scalability toward application in humans. In conclusion, this study sets the stage for clinically enabling toxicity and efficacy studies in a large animal model such as rabbit and ultimately first-in-human evaluation of this image-guided ablative procedure for BC risk reduction.

## Supplementary information

Supplementary information

## Data Availability

All data generated or analyzed during this study are included in this published article and its Supplementary Information files.

## Code availability

The underlying code for this study and training/validation datasets is not publicly available but may be made available to qualified researchers on reasonable request from the corresponding author.

## References

[1] Siegel, R. L., Miller, K. D., Wagle, N. S. & Jemal, A. Cancer statistics, 2023. *CA Cancer J. Clin.* **73**, 17–48 (2023). 36633525 10.3322/caac.21763

[2] Britt, K. L., Cuzick, J. & Phillips, K. A. Key steps for effective breast cancer prevention. *Nat. Rev. Cancer* **20**, 417–436 (2020). 32528185 10.1038/s41568-020-0266-x

[3] Padamsee, T. J., Wills, C. E., Yee, L. D. & Paskett, E. D. Decision making for breast cancer prevention among women at elevated risk. *Breast Cancer Res.* **19**, 34 (2017). 28340626 10.1186/s13058-017-0826-5 PMC5366153

[4] Slawson, S. H. & Johnson, B. A. Ductography: how to and what if? *Radiographics* **21**, 133–150 (2001). 11158649 10.1148/radiographics.21.1.g01ja15133

[5] Sheiman, L. S. & Levesque, P. H. The in’s and out’s of ductography: a comprehensive review. *Curr. Probl. Diagn. Radiol.* **45**, 61–70 (2016). 26163736 10.1067/j.cpradiol.2015.05.007

[6] Sapienza Passos, J., Dartora, V., Cassone Salata, G., Draszesski Malago, I. & Lopes, L. B. Contributions of nanotechnology to the intraductal drug delivery for local treatment and prevention of breast cancer. *Int. J. Pharm.* **635**, 122681 (2023). 36738808 10.1016/j.ijpharm.2023.122681

[7] Zaluzec, E. K. & Sempere, L. F. Systemic and local strategies for primary prevention of breast cancer. *Cancers* **16**, 248 (2024). 38254741 10.3390/cancers16020248 PMC10814018

[8] Kenyon, E. et al. Ductal tree ablation by local delivery of ethanol prevents tumor formation in an aggressive mouse model of breast cancer. *Breast Cancer Res.* **21**, 129 (2019). 31779648 10.1186/s13058-019-1217-x PMC6883550

[9] Chakravarty, S. et al. Tantalum oxide nanoparticles as versatile contrast agents for X-ray computed tomography. *Nanoscale* **12**, 7720–7734 (2020). 32211669 10.1039/d0nr01234c PMC7185737

[10] Kenyon, E. et al. X-Ray visualization of intraductal ethanol-based ablative treatment for prevention of breast cancer in rat models. *J. Vis. Exp*. **190**, e64042 (2022). 10.3791/64042 PMC9876732 36571406

[11] Kenyon, E. et al. Intraductal delivery and X-ray visualization of ethanol-based ablative solution for prevention and local treatment of breast cancer in mouse models. *J. Vis. Exp*. **182**, e63457 (2022). 10.3791/63457 PMC9613378 35435915

[12] Robertson, N. et al. Omniparticle contrast agent for multimodal imaging: synthesis and characterization in an animal model. *Mol. Imaging Biol.* **25**, 401–412 (2023). 36071300 10.1007/s11307-022-01770-w PMC9989039

[13] Wang, G. et al. Intraductal fulvestrant for therapy of ERalpha-positive ductal carcinoma in situ (DCIS) of the breast-a preclinical study. *Carcinogenesis* **40**, 903–913 (2019). 31046118 10.1093/carcin/bgz084

[14] Brock, A. et al. Silencing HoxA1 by intraductal injection of siRNA lipidoid nanoparticles prevents mammary tumor progression in mice. *Sci. Transl. Med.* **6**, 217ra212 (2014). 10.1126/scitranslmed.3007048 PMC5546412 24382894

[15] Chun, Y. S. et al. Intraductally administered pegylated liposomal doxorubicin reduces mammary stem cell function in the mammary gland but in the long term, induces malignant tumors. *Breast Cancer Res. Treat.* **135**, 201–208 (2012). 22752247 10.1007/s10549-012-2138-x PMC3478104

[16] Chun, Y. S. et al. Intraductal administration of a polymeric nanoparticle formulation of curcumin (NanoCurc) significantly attenuates incidence of mammary tumors in a rodent chemical carcinogenesis model: implications for breast cancer chemoprevention in at-risk populations. *Carcinogenesis* **33**, 2242–2249 (2012). 22831956 10.1093/carcin/bgs248 PMC3584967

[17] Murata, S. et al. Ductal access for prevention and therapy of mammary tumors. *Cancer Res.* **66**, 638–645 (2006). 16423990 10.1158/0008-5472.CAN-05-4329

[18] Okugawa, H. et al. Effect of perductal paclitaxel exposure on the development of MNU-induced mammary carcinoma in female S-D rats. *Breast Cancer Res. Treat.* **91**, 29–34 (2005). 15868429 10.1007/s10549-004-6455-6

[19] Sivaraman, L. et al. Effect of selective ablation of proliferating mammary epithelial cells on MNU induced rat mammary tumorigenesis. *Breast Cancer Res. Treat.* **73**, 75–83 (2002). 12083633 10.1023/a:1015227719105

[20] Stearns, V. et al. Preclinical and clinical evaluation of intraductally administered agents in early breast cancer. *Sci. Transl. Med.* **3**, 106ra108 (2011). 22030751 10.1126/scitranslmed.3002368 PMC3616888

[21] Yoshida, T. et al. Effective treatment of ductal carcinoma in situ with a HER-2- targeted alpha-particle emitting radionuclide in a preclinical model of human breast cancer. *Oncotarget* **7**, 33306–33315 (2016). 27119227 10.18632/oncotarget.8949 PMC5078096

[22] de Groot, J. S. et al. Intraductal cisplatin treatment in a BRCA-associated breast cancer mouse model attenuates tumor development but leads to systemic tumors in aged female mice. *Oncotarget* **8**, 60750–60763 (2017). 28977823 10.18632/oncotarget.18490 PMC5617383

[23] Kim, B. Y., Rutka, J. T. & Chan, W. C. Nanomedicine. *N. Engl. J. Med.* **363**, 2434–2443 (2010). 21158659 10.1056/NEJMra0912273

[24] DiCorpo, D. et al. The role of Micro-CT in imaging breast cancer specimens. *Breast Cancer Res. Treat.* **180**, 343–357 (2020). 32020431 10.1007/s10549-020-05547-z

[25] Hsu, J. C. et al. Nanoparticle contrast agents for X-ray imaging applications. *Wiley Interdiscip. Rev. Nanomed. Nanobiotechnol.* **12**, e1642 (2020). 32441050 10.1002/wnan.1642 PMC7554158

[26] Zhao, H. et al. Molecular imaging and deep learning analysis of uMUC1 expression in response to chemotherapy in an orthotopic model of ovarian cancer. *Sci. Rep.* **10**, 14942 (2020). 32913224 10.1038/s41598-020-71890-2 PMC7484755

[27] Sempere, L. F., Zaluzec, E., Kenyon, E., Kiupel, M. & Moore, A. Automated five-color multiplex co-detection of MicroRNA and protein expression in fixed tissue specimens. *Methods Mol. Biol.* **2148**, 257–276 (2020). 32394388 10.1007/978-1-0716-0623-0_17

[28] Lai, Y. E., Morhard, R., Ramanujam, N. & Nolan, M. W. Minimally invasive ethyl cellulose ethanol ablation in domesticated cats with naturally occurring head and neck cancers: six cats. *Vet. Comp. Oncol.* **19**, 492–500 (2021). 33583138 10.1111/vco.12687

[29] Mueller, J. L. et al. Optimizing ethyl cellulose-ethanol delivery towards enabling ablation of cervical dysplasia. *Sci. Rep.* **11**, 16869 (2021). 34413378 10.1038/s41598-021-96223-9 PMC8376953

[30] Nief, C. et al. Polymer-assisted intratumoral delivery of ethanol: preclinical investigation of safety and efficacy in a murine breast cancer model. *PLoS ONE* **16**, e0234535 (2021). 33507942 10.1371/journal.pone.0234535 PMC7843014

[31] Chelales, E. et al. Radiologic-pathologic analysis of increased ethanol localization and ablative extent achieved by ethyl cellulose. *Sci. Rep.* **11**, 20700 (2021). 34667252 10.1038/s41598-021-99985-4 PMC8526742

[32] Morhard, R. et al. Understanding factors governing distribution volume of ethyl cellulose-ethanol to optimize ablative therapy in the liver. *IEEE Trans. Biomed. Eng.* **67**, 2337–2348 (2020). 31841399 10.1109/TBME.2019.2960049 PMC7295656

[33] Morhard, R. et al. Development of enhanced ethanol ablation as an alternative to surgery in treatment of superficial solid tumors. *Sci. Rep.* **7**, 8750 (2017). 28821832 10.1038/s41598-017-09371-2 PMC5562881

[34] Sannier, K. et al. A new sclerosing agent in the treatment of venous malformations. Study on 23 cases. *Interv. Neuroradiol.* **10**, 113–127 (2004). 20587223 10.1177/159101990401000203 PMC3464441

[35] Dompmartin, A. et al. Radio-opaque ethylcellulose-ethanol is a safe and efficient sclerosing agent for venous malformations. *Eur. Radiol.* **21**, 2647–2656 (2011). 21822948 10.1007/s00330-011-2213-4

[36] Fujimichi, Y., Sasaki, M., Yoshida, K. & Iwasaki, T. Effects of irradiation on cumulative mortality in mice: shifting toward a younger age of death. *J. Radiat. Res.* **64**, 412–419 (2023). 36763980 10.1093/jrr/rrad006 PMC10036085

[37] Russo, I. H. & Russo, J. Developmental stage of the rat mammary gland as determinant of its susceptibility to 7,12-dimethylbenz[a]anthracene. *J. Natl Cancer Inst.* **61**, 1439–1449 (1978). 102856

[38] Paine, I. S. & Lewis, M. T. The terminal end bud: the little engine that could. *J. Mammary Gland Biol. Neoplasia* **22**, 93–108 (2017). 28168376 10.1007/s10911-017-9372-0 PMC5488158

[39] Hughes, K. Comparative mammary gland postnatal development and tumourigenesis in the sheep, cow, cat and rabbit: exploring the menagerie. *Semin. Cell Dev. Biol.* **114**, 186–195 (2020). 33082118 10.1016/j.semcdb.2020.09.010

[40] Schöniger, S., Degner, S., Jasani, B. & Schoon, H.-A. A review on mammary tumors in rabbits: translation of pathology into medical care. *Animals* **9**, 762 (2019). 31581718 10.3390/ani9100762 PMC6826878

[41] Falconer, I. R. The distribution of 131 I- or 125 I-labelled prolactin in rabbit mammary tissue after intravenous or intraductal injection. *J. Endocrinol.* **53**, 58–59 (1972). 5039234

[42] Fiddler, T. J., Birkinshaw, M. & Falconer, I. R. Effects of intraductal prolactin on some aspects of the ultrastructure and biochemistry of mammary tissue in the pseudopregnant rabbit. *J. Endocrinol.* **49**, 459–469 (1971). 5104551 10.1677/joe.0.0490459

[43] Fiddler, T. J. & Falconer, I. R. The effect of intraductal prolactin on protein and nucleic acid biosynthesis in the rabbit mammary gland. *Biochem. J.* **115**, 58P–59P (1969). 5360712 10.1042/bj1150058p PMC1185301

[44] Bourne, R. A., Bryant, J. A. & Falconer, I. R. Stimulation of DNA synthesis by prolactin in rabbit mammary tissue. *J. Cell Sci.* **14**, 105–111 (1974). 4856240 10.1242/jcs.14.1.105

[45] Chadwick, A. Detection and assay of prolactin by the local lactogenic response in the rabbit. *J. Endocrinol.* **27**, 253–263 (1963). 14079513 10.1677/joe.0.0270253

[46] Clark, A., Bird, N. K. & Brock, A. Intraductal delivery to the rabbit mammary gland. *J. Vis. Exp*. **121**, e55209 (2017). 10.3791/55209 PMC5408859 28362409

