# Supplementary material for: Tantalum oxide nanoparticles as versatile and high-resolution X-ray contrast agent for intraductal image-guided ablative procedure in rodent models of breast cancer
Source: Npj Imaging. 2024 Feb 19;2:3. doi: 10.1038/s44303-024-00007-5 (PMC11412094; doi:10.1038/s44303-024-00007-5)
Supplement: Supplementary file 1 — Supplementary information [file 44303_2024_7_MOESM1_ESM.pdf]

SUPPLEMENTAL MATERIALS

Supplemental figures

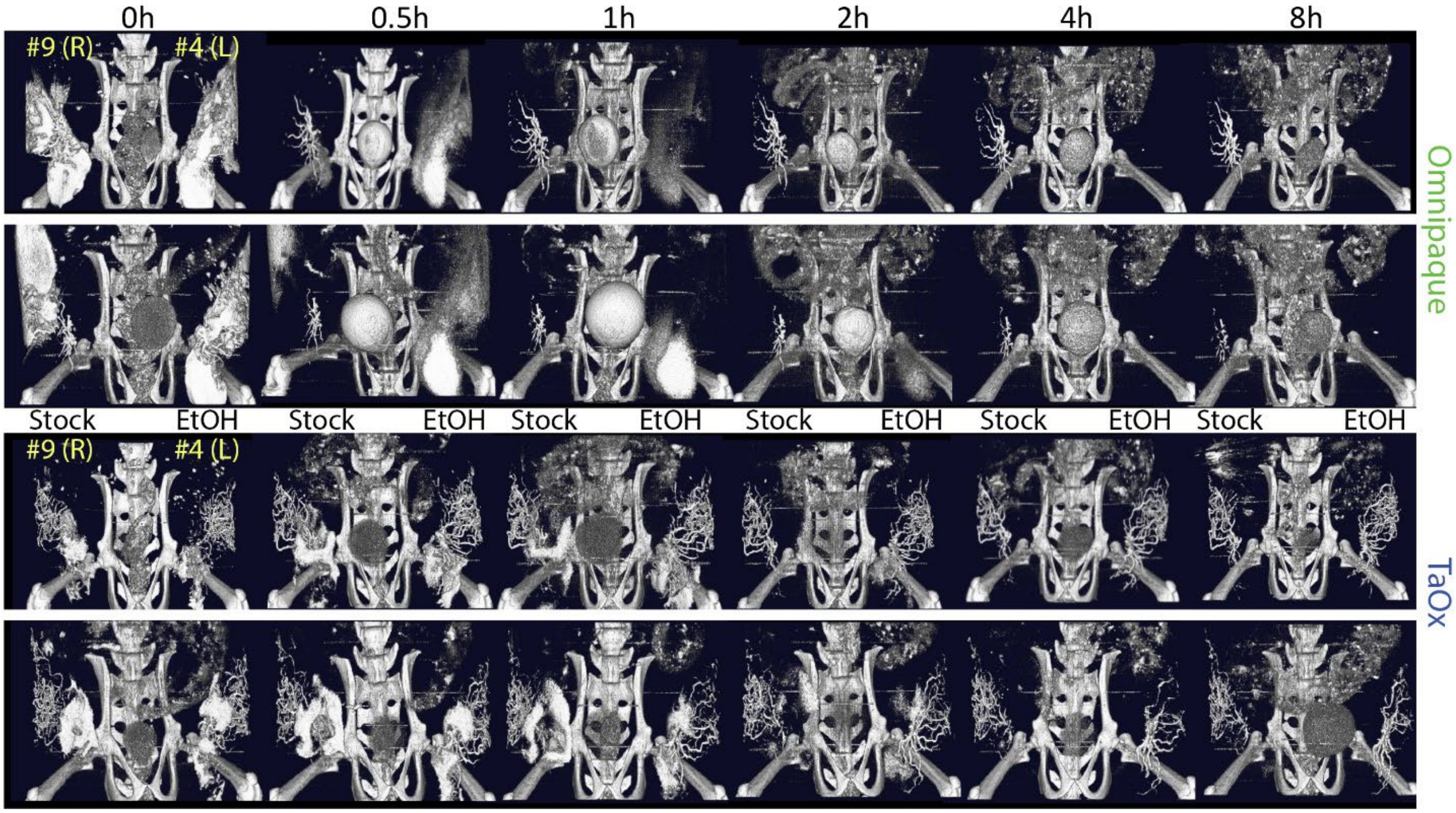

Figure S1. Short-term serial microCT imaging of the murine ductal tree with different contrast agents in different solutions. Abdominal mammary glands were injected with 40 μl of indicated contrast agent as stock solution (300 mg I/mL Omnipaque or 18 mg Ta/mL in PBS) or in 70% ethanol (EtOH, 90 mg I/mL Omnipaque or 18 mg Ta/mL). 3D reconstructions of lower body with PerkinElmer Quantum GX microCT software package include infused ductal trees (i.e., ipsi- and contralateral abdominal mammary glands), skeleton (i.e., lower spine, hip, and femurs), and internal organs (e.g., bladder, hyperchromatic bladder is indicative of Omnipaque's systemic clearance). 3D reconstruction from the same animals are shown at different imaging time points from immediately after to 8 hrs after infusions.

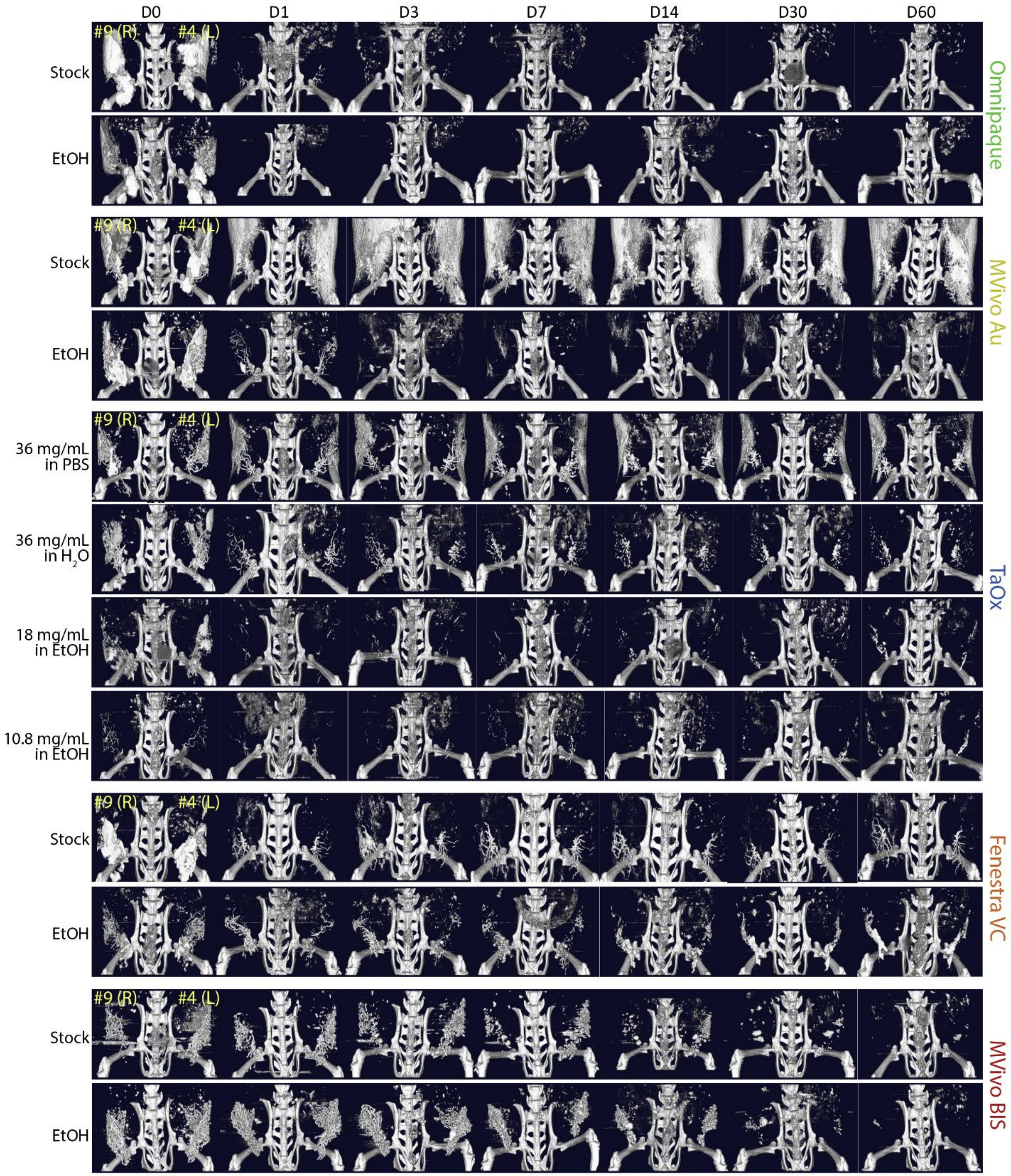

Figure S2. Long-term serial microCT imaging of the murine ductal tree with different contrast agents in different solutions. Abdominal mammary glands were injected with 40 μl of indicated contrast agent as stock solution: Omnipaque (300 mg I/mL), MVivo Au (200 mg Au/mL), TaOx (36 mg Ta/mL), Fenestra VC (50 mg I/mL), MVivo BIS (150 mg Bis/mL); or in 70% EtOH: Omnipaque (90 mg I/mL), MVivo Au (60 mg Au/mL), TaO_x

(10.8 mg Ta/mL), Fenestra VC (15 mg I/mL), MVivo BIS (45 mg Bis/mL). 3D reconstructions of lower body with PerkinElmer Quantum GX microCT software package include infused ductal trees (i.e., ipsi- and contralateral abdominal mammary glands), skeleton (i.e., lower spine, hip, and femurs), and internal organs (i.e., skin, hyperchromatic subcutaneous layer is indicative of MVivo Au's regional accumulation). 3D reconstructions from the same animals are shown at different imaging time points from immediately after (D0) to 60 days (D60) after infusions.
